# Mississippi Valley Medical Association

**Published:** 1890-11

**Authors:** 


### MISSISSIPPI VALLEY MEDICAL ASSOCIATION.

### SIXTEENTH ANNUAL MEETING HELD IN LOUISVILLE, KENTUCKY, OCTOBER 8, 9, 10, 1890.

#### OCTOBER 8TH—FIRST DAY—MORNING SESSION.

The Association met in Liederkrans Hall, and was called to order by the President, Dr. J. M. Mathews, of Louisville, at 10 A. M.

Prayer was offered by the Rev. Mr. Jones, of Louisville.

Dr. I. N. Bloom, of Louisville, Chairman of the Committee of Arrangements, made his report.

The first paper read was by Dr. Frank Woodbury, of Philadelphia, Pa., entitled

INFECTIOUS DYSPEPSIA AND ITS RATIONAL TREATMENT BY THE ANTISEPTIC METHOD, in which he said it was proper to state, at the outset, that our present consideration of the subject is limited to dyspepsia solely as related to the stomach, no reference was attempted, or intended to be made, to intestinal indigestion or to the so-called intestinal dyspepsia.

With reference to the pathology of dyspepsia, he considered it at least as much entitled to recognization as a distinct disea se in the present unsettled condition of medical nomenclature, as consumption or chorea. Like them, it is characterized clinically by manifestations of nervous disorder; so that Cullen was not very far wrong in considering dyspepsia as a neurosis under the class of Adynamiæ. Like pulmonary phthisis, also, its most marked symptoms are produced (the author believes) by the ab, sorption of the products of parasitic micro-organisms

Of late years the science of bacteriology has made wonderful advance, and especially in the department of bacterial parasiticism or infection, and its relation to disease.

Abelous, a recent investigator of this subject, found 16 species existing normally in his own stomach, of which 2 were micrococci, 13 bacilli and 1 vibrio. The presence of saprogenic microbes in the stomach, therefore, being constant and not incompatible with health, it becomes necessary to inquire why fermentation or putrefaction of the food does not occur after every meal? In other words, how is practical antisepsis obtained by natural process? Three things are to be considered in this connection; (1) the food, (2) the digestive fluids, and (3) the physical conditions attending the act of digestion.

Dr. Woodbury summarized as follows:

Laborious, painful and imperfect digestion occurring habitually, when not symptomatic of other disease, constitutes dyspepsia; and when accompanied by fermentation of the contents of the stomach and general toxic symptoms, the result of microbian development, it may properly be called infectious dyspepsia.

The disorder is sufficiently prevalent, and gives rise to enough discomfort and actual suffering in its victims not only to deserve our serious consideration, but also to enlist our best therapeutic skill in their behalf. The excessive growth of micro-organisms during digestion is favored by slow movements of the stomach, and by defective quantity or quality of the gastric juice. Acid dyspepsia, or sour stomach, may be due to excessive secretion of hydrochloric acid (rarely), but is generally caused by lactic acetic or butyric fermentation, due to the presence of appropriate forms of bacteria in the stomach. The object of treatment of infectious dyspepsia is to prevent the excessive development of micro-organisms during the digestion of food. This is sought to be accomplished (1) by the use of articles of diet which are not in a fermenting condition nor readily fermentable ; (2) by adopting such hygienic and tonic measures as will invigorate the bodily powers and especially bring the gastric juice up to its normal standard of quality and quantity, and increase the muscular power of the stomach, and (3) by local antiseptic treatment, including the administration of drugs which retard fermentation, and especially by lavage or irrigation of the stomach with weak disinfectant solutions, or simply recently boiled water.

Dr. John N. Hollister, of Chicago, contributed a paper on

HELP AND HINDRANCE TO MEDICAL PROGRESS.

He said the possibility of progress was conditioned upon the present imperfection of present attainment; results are dependent upon our abilities, upon our methods, and upon the obstacles to be overcome. The profession must command a much higher average of native talent; that talent must receive a much higher grade of culture; and the present methods of research, on the part of the profession, must be greatly modified and improved.

Dr. I. N. Love, of St. Louis, Missouri, in a paper entitled

COFFEE, said that his experience for five or six years past is strongly in favor of taking a cup of strong, black coffee, without cream or sugar, sandwiched in between two glasses of hot water, before rising every morning at least one hour before breakfast. The various secretions are stimulated, the nervous force is aroused, an hour later a hearty meal is enjoyed, and the day's labor is commenced favorably, no matter how the duties of the day and night preceding may have drawn upon the nervous system. Another cup at four in the afternoon is sufficient to sustain the energies for many hours. In this way the full effect is secured. The stimulant devotes itself strictly to business, none of it is lost, and if the proper diet be taken at the proper time, and the ideal diet for those who make large drafts upon their nervous system and expect to have them honored, is hot milk. If the above regimen be followed, and accompanied by at least eight hours of sleep out of every twenty-four hours, the capacity for work is almost unlimited.

Dr. Geo. Hulbert, of St. Louis, Missouri, read an interesting paper on

MECHANICAL OBSTRUCTION IN DISEASES OF THE UTERUS, and submitted the following conclusions:.

1. That in the natural or normal order of things do we find the uterus, in form and structure, endowed with a power and capacity for the performance of the function, menstruation, far in excess of any legitimate demand, to the extent that, with a one-fourth inch diameter of canal at the sphincters, the excess equals 7,724.8 times the demand, and with a one thirty-second inch diameter, the excess equals 120.7 times the requirement.

2. That in the pathological conditions, considered as essential for mechanical obstruction, do we find that the conservation of force is capable, and does so regulate conditions that the capacity is not abolished, but persistent in an eminent degree, so that in the presence of the normal physiological energy the function is accomplished save in only one emergency, that of total annihilation of the normal state, namely, atresia.

3. That the phenomena considered as attendant and dependent upon mechanical obstruction, are not due to the forcible expulsion of retained fluids through the uterine canal, but are resident and produced within the tissues, and are dependent upon disturbed rhythm of physiological forces, evolved through abnormal enervation, muscular action and circulation.

4. That the demand upon the uterus, for the passage of blood clots, membranes, mucous plugs, uterine sounds, sponge tents, uterine dilators, etc., in order that the diagnosis of mechanical obstruction may be made, is not only vicious in the extreme, but irrational, illogical, and unscientific.

5. That the correct and rational interpretation of the testimony offered, by systematology, pathology and therapeutics, removes mechanical obstruction from the domain of gynecology, as a demonstrable fact, save in "atresia uteri."

FIRST DAY—AFTERNOON SESSION.

The Association was called to order at 3 p. m. by first Vice-President, Dr. C. R. Early, of Rigeway, Pa.

Dr. R. Stransberry Sutton, of Pittsburgh, Pa., made (by invitation) some remarks on the surgical treatment uterine fibroids, and exhibited specimens.

Dr. William Porter, of St. Louis, Missouri, contributed a paper entitled

PROFESSOR FLINT'S DOCTRINE OF THE SELF-LIMITATION OF PHTHISIS.

Dr. A. B. Thrasher, of Cincinnati, O., read a paper on

COUGH, ITS RELATION TO INTRA-NASAL DISEASE.

He said cough is a reflex phenomenon due to the irritation of a nerve fibre in the air-tubes, larynx, pharynx, nose, ear, stomach, etc.

A normal cough is for the purpose of freeing the air tract from some foreign body. Irritation of the upper part of the trachea and the ventricles of morgagni most frequently produces cough. An irritation in many others locations may be referred by the sensory centres to this region, and thus give rise to cough. Inflammation of the cavernous bodies of the nose, or of the adjacent septum, has been known to give rise to a distressing cough, and has been mistaken for evidence of tubercular disease. This is more apt to occur in a person of neurotic temperament. The cough due to nasal disease may sometimes be recognized by its metallic ring and the absence of expectoration. It can, as a rule, be provoked at will, by touching the irritable spot in the nose with a silver probe. Dr. Thrasher recited three cases illustrative of nasal cough from his private practice. In Case I. there were no subjective symptoms of nasal disease. The cough had been present for three months and was not benefited by the usual cough mixtures. The lower turbinate was found to be hypertrophic, and touching it with a probe provoked violent coughing. The cautery was applied, and in three days the patient ceased to cough.

CASE II. A young lady had been coughing violently for three months. She referred the irritation to the throat, which had been pencilled and sprayed for some time with no relief. Touching the posterior extremities of either lower turbinate produced violent coughing. Treatment as in Case I, with good result in two months.

CASE III. Cough had been present six months and was not benefited by constitutional or local treatment. The seat of the trouble was found to be in the left middle and right lower turbinate. Treatment similar to other cases was followed by cessation of cough within a month.

A paper entitled Therapeutic Uses of Cardiac Sedatives in Inflammation, by Dr. Hobart Amory Hare, of Philadelphia, Pa., was read by title in the absence of the author.

Dr. A. H. Ohmann-Dumesnil, of St. Louis, Missouri, reported a case of rhinothyma—operation ?

---

FIRST DAY—EVENING SESSION.

Dr. John A. Wyeth, of New York City, delivered the public address, taking for his subject THE MEDICAL STUDENT.

The immense hall was literally packed with people, and the members of the Mississippi Valley Medical Association—a great many of them—who came to hear the lecture were turned away, the students of the Louisville University and the laity having taken possession of nearly all the seats, thus literally freezing the members out. The address was listened to very attentively, and Dr. Wyeth received applause several times during its delivery.

He said, in the course of his remarks, that the *first* or preliminary stage of a medical student's life is his preparatory or academic life; the *second* his medical college life; the *third* his post-graduate or practical life, and it extends from the day he leaves his alma mater until usefulness ceases. In the acquirement of a practical training three ways were open, and in order or preference they were:

1. Service as interne, preferably for a term of two years in a general hospital.

2. Service in some post-graduate institution where all departments of practical medicine are taught by teachers especially trained in their respective branches.

3. Service as assistant to one or more well-qualified practitioners in general medicine.

OCTOBER 9TH—SECOND DAY—MORNING SESSION.

Dr. Joseph Ransohoff, of Cincinnati, Ohio, read a paper on

CHRONIC DISEASES OF THE JOINTS.

Dr. H. C. Dalton, of St. Louis, Missouri, contributed a paper entitled

CASES OF PENETRATING STAB WOUNDS OF THE ABDOMEN, LAPAROTOMY—RESULTS.

Dr. Dalton reported six cases of laparotomy in which there was visceral injury. There was one death and five recoveries.

CASE I. Was a wound of the descending colon and ileum; recovery, E. T., colored, aet. sixteen, was admitted to the city Hospital, of St. Louis, July 23, 1890. Patient was stabbed two hours previously with long bladed knife. Wound at free extremity of the 12th rib, several inches protruded; general condition excellent; pulse 62, respiration 23, temperature 100. Incision was made in the left linea semilunaris; blood and fecal matter found in the cavity; there were two holes in the descending colon, one in the ileum, which were closed with continued iron-dyed silk sutures. Discharged from hospital eleven days after admission.

CASE II. Stab wound of liver and intestines; recovery, A. V., aet. 21, admitted August 21, 1890, received three stab wounds an hour and a half before admission, one was an inch below the costal border, four inches to the left of median line; three inches of omentum protruded; second was an inch above, and two inches to the right of the umbilicus; third in the seventh interspace in the right axillary line. The wound of the jejunum was closed by an interrupted silk suture. Four inches of the seventh rib were resected, the diaphragm split up three inches, and the wound in the liver closed by one catgut suture. Diaphragmatic and cutaneous wounds closed by continuous catgut suture. Patient's temperature arose to 102 on the second day, after which he recovered rapidly.

Dr. Dalton laid particular stress upon the necessity of following the wounds to the bottom and making ocular inspection of the same, and severely condemned the method of trusting to the introduction of the finger in the *tactus eruditus*

He deprecated depending implicitly upon Senn's hydrogen gas test on account of its fallibility.

These cases were generally discussed, and the doctor highly complimented on his good results.

Dr. M. T. Scott, of Lexington, Kentucky, reported a case of

GUN SHOT WOUND OF THE INTESTINE, in which there were four perforations by a large bullet, various complications, and complete recovery following laparotomy.

Dr. J. B. Murdoch, of Pittsburgh, Pa., contributed a paper on

TORSION OF ARTERIES AS A MEANS FOR THE ARREST OF HEMORRHAGE.

He said there is no subject of greater interest to the practical surgeon than the arrest of hemorrhage. This remark is equally true whether the hemorrhage comes from a wound accidentally inflicted, or one made intentionally, by the surgeon's knife.

There are two methods by which the torsion may be applied: (1) Limited torsion, and (2) free torsion. In the first method, two pairs of forceps are required. The first pair grasps the vessel at its cut extremity, and pulls it from the sheath. It is then seized by the second pair at a point from one-half an inch to an inch above the cut extremity of the artery; this second pair being held at right angles to the long axis of the vessel. The first pair is then given three or four sharp turns.

By the second method (free torsion) only one pair of forceps is required. It is the one recommended by Mr. Bryant as not being so likely to injure the external coat of the artery. And this is the method which was adopted in the cases which are given herewith.

The following table shows the number of arteries divided in cases of amputation, where torsion has been resorted to for the arrest of hemorrhage, at the Western Pennsylvania Hospital:

| | | |
|---|---|---|
| Femoral | 116 | times. |
| Popliteal | 18 | " |
| Axillary | 18 | " |
| Anterior tibial | 317 | " |
| Brachial | 81 | " |
| Posterior | 317 | " |
| Radial | 45 | " |
| Ulnar | 45 | " |

Dr. Murdoch said in conclusion that the advantages of torsion as compared with ligation are: (1) The greater facility with which it can be applied. (2) Torsion is a safer method, being less liable to be followed by secondary hemorrhage. (3) Healing is facilitated because the wound is free from any irritating or foreign body.

Dr. G. Frank Lydston, of Chicago, Illinois, exhibited the skulls of a number of the most notorious criminals known in history, and made some very instructive remarks relative to their peculiarities, shape, size, etc.

### SECOND DAY—AFTERNOON SESSION.

Dr. W. P. King, of Kansas City, Missouri, contributed a paper entitled

### WIRING THE SEPARATED SYMPHYSIS-PUBIS, SUPPLEMENTED BY A NOVEL PELVIC CLAMP.

He reported a case of separation of the symphysis-pubis, with fracture of the interposed fibro-cartilages, fracture of the descending ramus of the pubis, with deep lacerations of the surrounding soft parts, and he referred particularly to the methods resorted to in order to support the pelvis and reinforce the stitches after the pubis had been wired together.

The case suggested the following points:

1. The operation of wiring in a case of separation of the symphysis-pubis so completely coaptates the parts that it would seem that scarcely any other method of dealing with this condition can be equal to it.

2. The manner of applying the plaster of Paris support in the first place, with the use of the water-bag to make an arch under which to dress the wounded parts, is new and original so far as the author knows, and it is a method that may be adopted and easily practiced by any one who knows how to use plaster of Paris.

3. The steel hip clamp, as a permanent support in separation of the pubis, is also new, so far as he knew, and is a means that may be adopted with benefit in any case of fracture of the pelvis wherein immobilization of the fractured part will contribute to the comfort of the patient and to the union of the fracture.

Dr. C. H. Hughes, of St. Louis, Missouri, read a very elaborate and profound paper entitled Psychopathic Sequences of Hereditary Alcoholic Entailment, which was followed by a paper on Urea and Serous Membranes, by Dr. C. S. Bond, of Richmond, Ind.

Dr. Arch. Dixon, of Henderson, Ky., read a paper on Inguinal Colotomy.

Dr. Emory Lanphear, of Kansas City, Missouri, read a paper on

HYPNOTISM IN ITS RELATION TO SURGERY, and reported cases.

He reported a case of double talipes in which the subject had chronic Bright's disease which contraindicated the use of ether, and at the same time he had an organic heart trouble, which prevented the safe use of chloroform. The patient wanted to be operated upon, but Dr. Lanphear hesitated to give the ordinary anæsthetic, and so hypnotized him. Contrary to the generally accepted idea that at the first seance a sufficient degree of anæsthesia cannot be produced to perform an operation, the doctor got a sufficient degree of anæsthesia by suggestion, by which he performed the operation for talipes, and the patient lay upon the table as fixed and immovable as marble during the whole operation.

In another case, which he reported by permission of Dr. Shaw, of St. Louis, the patient suffered from Jacksonian epilepsy, due to a brain tumor. He was hypnotized. The scalp cut, an inch and a half trephine used, the dura mater opened, and a brain tumor weighing one ounce and a half removed. The bone was replaced and the operation completed, occupying an hour without the use of chloroform or ether, the patient being simply under the hypnotic influence.

Dr. Theo. Potter, of Indianapolis, Ind., presented a paper entitled "Certainty in the Diagnosis of Tuberculosis," in which he said there was no important chronic diseases in which our opinions, as physicians, are more frequently sought, are more weighty, and more subject to present and future criticism, than tuberculosis.

The lack of specific curative treatment, and of any great tendency to self-limitation, after once well under headway; the destruction of tissue; the existence of subtile predisposing as well as exciting elements; the establishment of the vicious circle, including the organs and channels of nutrition, the strange, and often persistent, delusion of hope, and finally the possibility of arrest or real cure; these factors call in a peculiar way for early treatment. But this must depend upon early diagnosis.

In spite of the constant progress from Lannec and Flint there is no one sign, and no combination of signs, which is absolute. There is always some uncertainty, and in the early or unusual cases we are, and often long remain, uncertain. These are the golden weeks and months. But now, with the new light of the present added to the knowledge of the past, we are able to make the diagnosis in the great majority of cases, not only early but with absolute certainty.

### THE HYPODERMIC USE OF ARSENIC.

Dr. Harold N. Moyer, of Chicago, Ill., read a paper on this subject. He said the hypodermic use of Fowler's solution has been recommended by various writers, among others, Hammond, who claimed that the dose which could be administered in this way was much greater than could be safely administered by the mouth, Hammond having given as high as 50 drops of Fowler's solution as an initial dose. Again, he has often carried the amount given by the mouth to the utmost bounds of prudence till the eyes were puffed and vomiting was almost incessant, and then has continued the arsenic in larger doses, by the hypodermic injection, with the result of the cessation of all gastric symtoms and the cure of the disorder.

In a case of chorea, female, æt. 14, the patient was placed immediately upon the hypodermic, beginning with three minims of the 5 per cent. solution, and increasing every second day, until three weeks after beginning treatment, she was receiving 13 minims of the solution at each injection, with an arsenic equivalent of about 36 minims of Fowler's solution. At the ninth injection she was discharged cured.

In the case of a woman who presented herself at the clinic in Rush Medical College with an enormous lymph adenoma of the side of the neck, after a few deep injections into the glandular mass it began rapidly to diminish, when it had lessened one-half the patient ceased attending, and further results could not be noted.

Dr. Moyer's observation is in accord with numerous writers, who have reported equally good results from the use of Fowler's solution in various forms of glanular enlargement, passing under the terms, lymphoma, lymph adenoma, Hodgkin's disease.

In conclusion Dr. Moyer said that the action of arsenic given under the skin, if it have any virtue, must certainly be greater than when taken by the stomach. Thrown into the cellular tissue in the form of a feeble alkaline and readily soluble salt it is at once absorbed by the blood and carried to all the tissues administered in this way.

OCTOBER 10TH—THIRD DAY—MORNING SESSION.

Dr. H. O. Walker, of Detroit, Mich., read a paper on

PERINEAL CYSTOTOMY VERSUS SUPRA-PUBIC CYSTOTOMY.

He said in the choice of method of operation we should be governed (1) as to its safety; (2) as to its simplicity of performance; (3) as to its rapidity of result; (4) as to its general applicability in the majority of cases. Dr. Walker reported several cases in which he resorted to the perineal method.

The treatment of enlarged prostate with cystitis is eqally efficacious by the perineal section and drainage, in furtherance of which he reported a very interesting case.

The perineal method of reaching the bladder is the oldest known to us, although numerous modifications have been made since the haphazard "cut on the gripe" for stone was first done. For the removal of stone litholapaxy undoubtedly stands pre-eminent, and can be done upon subjects from three years of age upwards, yet there are numerous restrictions to this method, such as stricture of the urethra, large sized stone, an enormous prostate, etc. There can be no question, said the author, when cutting has to be done, that the medio-bilateral method presents the best advantages.

In looking up the literature at his command upon supra-pubic operations since 1883, Dr. Walker finds, in the records of between three and four hundred operations, an average mortality of 30 per cent. A few operators have had a series of cases, ranging from three to ten, without a death, the most remarkable record in this respect being that of the distinguished surgeon, Dr. Hunter McGuire—twenty-one operations with but a single death. By the perineal method he finds a mortality of but five, six and seven per cent., rarely ever going beyond nine per cent.

Dr. Edwin Walker, of Evansville, Indiana, read a paper entitled

TWO CASES OF TUBAL PREGNANCY.

The first case was Mrs. E. S., aged twenty-seven, married four years, sterile. She had a history of uterine and tubal trouble before marriage. Since marriage patient has been an invalid, suffering pain in right groin. Menses always irregular, often missing a month or two. She was unwell June 29, 1890, but in July missed her menstrual period. A few days later she began to suffer from a severe pain in the right groin. August 1st, a sanguinous flow began, and continued to the time of operation. Examination under ether revealed a soft tumor the size of the fist to the right and behind the uterus. August 17th, abdomen was opened and the right tube, which was very large, found ruptured and a large amount of clotted blood in the pelvis; fœtus not found; abdomen irrigated with hot water; glass drainage tube used. Some vomiting and pain, but recovery ensued without a bad symptom. Drain removed on the third and sutures on the twelfth day. Highest temperature was 101.1.

The author thinks that the present status of the question is, that with such a class of symptoms as presented in this and other cases, laparotomy is the safest procedure to adopt.

Dr. William T. Belfield, of Chicago, Illinois, in a contribution entitled

RESUME OF EXPERIENCE TO DATE ALL OVER THE WORLD IN THE VARIOUS OPERATIONS FOR CYSTITIS FROM PROSTATIC HYPERTROPHY, collected one hundred and thirty-three cases of operations upon the hypertrophied prostate, including eight of his own as follows: Forty-one by perineal incision, mortality nine per cent; eighty-eight by supra-pubic cystotomy, mortality sixteen per cent; four by combined perineal and supra-pubic incision, none fatal.

In fifty-six of these cases the essential facts before and after operation are furnished; they had been the subjects of cystitis and dependent upon the catheter for periods varying from one to ten years. In all the cystitis was cured; in thirty-eight (two-thirds) voluntary urination was restored and continued during the time of observation, six months to two and a half years; in eighteen this function was not recovered.

Fifteen of these fifty-six cases were complicated with stone; excluding these, since it might be objected that the cure resulted rather from the calculus extraction than from the prostatic operation, there remain forty-one cases of uncomplicated prostate operations; of these thirty-two (four-fifths) recovered the power of urination, in nine this ability was not recovered.

Dr. Edwin Ricketts, of Cincinnati, Ohio, read a paper on The Difficulty in Diagnosing a Twisted Ovarian Pedicle in Uterine Myoma; Dr. David Barrow, of Lexington, Kentucky, one entitled Three Cases of Intestinal Obstruction, with remarks, and Dr. R. R. Kime, of Petersburg, Indiana, one entitled Extra-Uterine Pregnancy, with report of a case of four years and three months duration, complicated with Entero-Uterine Fistula.

### THIRD DAY—AFTERNOON SESSION.

Dr. Seaton Norman, of Evansville, Indiana, contributed a paper entitled

TREATMENT OF ORGANIC STRICTURE OF THE MALE URETHRA

in which he said in the practice of urethral surgery the operator cannot be too emphatically impressed with the fact of the requisite tenderness and sensitiveness of the urethra, and the employment of the slightest amount of force in the introduction of an instrument should be regarded as a relic of barbaric surgery When commencing the treatment by gradual dilatation, in sensitive patients, he always produces local anæsthesia by the injection of twenty to thirty minims of a four per cent. solution of hydrochlorate of cocaine.

Relative to internal urethrotomy, he believes that, when it is properly and thoroughly executed, and special care is exercised to maintain the patentcy of the canal until the wound is entirely healed, reconstr ction is of rare occurrence. Authority is divided in regard to the performance of internal urethrotomy in the bulbous and membranous urethra. Judging from results obtained by Harrison, the combination of external and internal urethrotomy offers encouragement for the permanent cure of stricture. Dr. Norman has performed external urethrotomy without a guide only three times, and his results, as regards the non-recurrence of contraction, have been entirely satisfactory. External urethrotomy with a guide is a simple operation; can be with facility and rapidity performed, and promises more satisfactorily ultimate results than internal urethrotomy performed in the deep urethra.

Of the various scales that have been proposed for urethral instruments, only the French system, in the opinion of Dr. Norman, is worthy of consideration. To have urethrotomes graduated in millimetres—and all of them with which the author is acquainted, are so manufactured—and the sounds corresponding to the English, or any other scale, is, in his judgment, a manifest absurdity.

Dr. L. S. McMurtry, of Louisville, Kentucky, made some impromptu remarks on

THE APPLICATION OF ANTISEPTIC METHODS IN MIDWIFERY PRACTICE.

He said many practitioners can remember the time when they heard that the wards of certain hospitals were closed and undergoing renovation because puerperal fever had become epidemic in such institutions. The hospital to-day is the safest place in which a woman can be confined. A few years ago, led by Fordyce Barker, we were taught that puerperal fever was an entity, a distinct fever, dependent upon a separate *materies morbi*, just the same as malarial fever is an entity. To-day we know that puerperal fever, so-called, is a septic peritonitis, just the same as when a women becomes infected after abdominal section, or after wounds of the peritoneum from any cause, or from infection of the endometrium, and through the Fallopian tubes to the peritoneum. A woman after labor is a wounded woman. She has undergone certain physiological processes; she has received certain injuries in the process of labor which opened the lymphatic channels by which she may have become infected from without. There is no such thing, said the speaker, as a woman having a peritonitis unless she is infected from without.

To prevent this infection the vagina should be sterilized, the bed surgically clean, the examining finger clean, the nurse clean, and the atmosphere as approximately aseptic as it is possible to make it, etc.

The following papers were also read: The Advantages of Attending Medical Societies and of Reading Medical Journals, by Dr. T. B. Greenley, of West Point, Ky.; Internal Urethrotomy, with Cases, by Dr. J. V. Prewitt, of West Point, Ky.; Was it Relapsing Fever? by A. D. Barr, M. D., Calamine Springs, Ark.; Some Remarks on the Prevention of Myopia, by Dr. Francis Dowling, of Cincinnati, O.

OFFICERS FOR 1891.

President—Dr. C. H. Hughes, St. Louis, Mo.
First Vice-President—Dr. John H. Hollister, Chicago, Ill.
Second Vice-President—Dr. S. S. Thorn, Toledo, O.
Secretary—Dr. E. S. McKee, Cincinnati, O.
Place of meeting, St. Louis, Missouri, third Wednesday in October, 1891.

